# 1,4-Diazo­niabicyclo­[2.2.2]octane hexa­aqua­magnesium bis­(sulfate)

**DOI:** 10.1107/S1600536811005368

**Published:** 2011-02-19

**Authors:** Wen-Ni Zheng

**Affiliations:** a Ordered Matter Science Research Center, College of Chemistry and Chemical Engineering, Southeast University, Nanjing 210096, People’s Republic of China

## Abstract

In the title compound, (C_6_H_14_N_2_)[Mg(H_2_O)_6_](SO_4_)_2_, the Mg^II^ ion, lying on an inversion center, is coordinated by six water mol­ecules in a slightly distorted octa­hedral geometry. The 1,4-diazo­niabicyclo­[2.2.2]octane cation is located about a twofold rotation axis. Inter­molecular N—H⋯O and O—H⋯O hydrogen bonds link the cations and the anions into a three-dimensional network.

## Related literature

For the properties and applications of amide salt compounds, see: Fu *et al.* (2007 ▶, 2008 ▶, 2009 ▶); Fu & Xiong (2008 ▶).
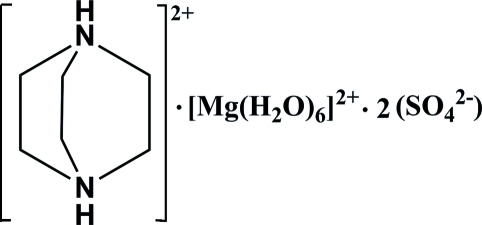


## Experimental

### 

#### Crystal data

(C_6_H_14_N_2_)[Mg(H_2_O)_6_](SO_4_)_2_
*M* *_r_* = 438.74
Monoclinic, 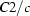

*a* = 14.968 (3) Å
*b* = 9.1860 (18) Å
*c* = 14.334 (3) Å
β = 117.12 (3)°
*V* = 1754.2 (8) Å^3^
*Z* = 4
Mo *K*α radiation
μ = 0.41 mm^−1^
*T* = 298 K
0.40 × 0.30 × 0.20 mm

#### Data collection

Rigaku SCXmini CCD diffractometer
Absorption correction: multi-scan (*CrystalClear*; Rigaku, 2005 ▶) *T*_min_ = 0.89, *T*_max_ = 0.95
8747 measured reflections
2014 independent reflections
1839 reflections with *I* > 2σ(*I*)
*R*_int_ = 0.022

#### Refinement

*R*[*F* ^2^ > 2σ(*F* ^2^)] = 0.065
*wR*(*F* ^2^) = 0.169
*S* = 1.26
2014 reflections
116 parameters
H-atom parameters constrained
Δρ_max_ = 1.30 e Å^−3^
Δρ_min_ = −1.08 e Å^−3^

### 

Data collection: *CrystalClear* (Rigaku, 2005 ▶); cell refinement: *CrystalClear*; data reduction: *CrystalClear*; program(s) used to solve structure: *SHELXS97* (Sheldrick, 2008 ▶); program(s) used to refine structure: *SHELXL97* (Sheldrick, 2008 ▶); molecular graphics: *SHELXTL* (Sheldrick, 2008 ▶) and *DIAMOND* (Brandenburg, 1999 ▶); software used to prepare material for publication: *SHELXTL*.

## Supplementary Material

Crystal structure: contains datablocks I, global. DOI: 10.1107/S1600536811005368/hy2403sup1.cif

Structure factors: contains datablocks I. DOI: 10.1107/S1600536811005368/hy2403Isup2.hkl

Additional supplementary materials:  crystallographic information; 3D view; checkCIF report

## Figures and Tables

**Table 1:** Hydrogen-bond geometry (Å, °)

*D*—H⋯*A*	*D*—H	H⋯*A*	*D*⋯*A*	*D*—H⋯*A*
N1—H1⋯O3^i^	0.91	1.87	2.734 (3)	157
O1*W*—H1*WA*⋯O3^i^	0.85	1.90	2.751 (3)	179
O1*W*—H1*WB*⋯O1^ii^	0.85	2.01	2.835 (3)	165
O2*W*—H2*WA*⋯O2	0.85	2.00	2.809 (3)	158
O2*W*—H2*WB*⋯O2^iii^	0.85	1.83	2.674 (3)	173
O3*W*—H3*WA*⋯O4^iv^	0.85	1.85	2.694 (3)	170
O3*W*—H3*WB*⋯O1^iii^	0.85	1.92	2.769 (3)	177

Symmetry codes: (i) 
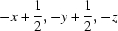
; (ii) 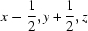; (iii) 
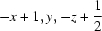
; (iv) 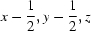.

**Fig. 1.**
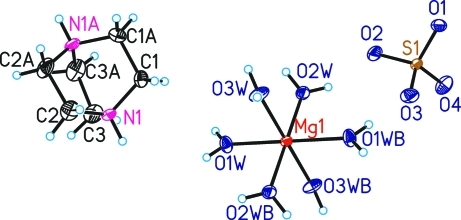
The asymmetric unit of the title compound. Displacement ellipsoids are drawn at the 30% probability level. [Symmetry codes: (A) -x, y, 0.5-z; (B) 0.5-x, 0.5-y, -z.]

**Fig. 2.**
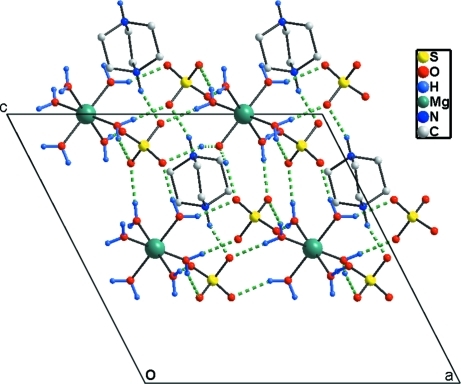
The crystal packing of the title compound, showing the three-dimensional hydrogen-bonded network. H atoms not involved in hydrogen bonds (dashed lines) have been omitted for clarity.

## supplementary crystallographic information

### Comment

Salts of amide have attracted more attention as phase transition dielectric materials for their applications in micro-electronics and memory storage (Fu *et al.*, 2007, 2008, 2009; Fu & Xiong, 2008). With the purpose of obtaining phase transition crystals, the interactions of 1,4-diazabicyclo[2.2.2]octane with various metal ions have been studied and we have elaborated a serie of new materials with this organic molecule. In this paper, we describe the crystal structure of the title compound.

The asymmetric unit is composed of an SO_4_^2-^ anion, half 1,4-diazoniabicyclo[2.2.2]octane cation and half [Mg(H_2_O)_6_]^2+^ cation. (Fig. 1). The Mg^II^ ion, lying on an inversion center, is in a slightly distorted octahedral geometry formed by six O atoms from the water molecules. The [Mg(H_2_O)_6_]^2+^ cation possesses typical Mg—O bond lengths [2.035 (2)–2.086 (2) Å], while the O—Mg—O bond angles [88.90 (8)–91.86 (9)°] indicating some distortion from a regular octahedron.

In the crystal, the interionic hydrogen bonds are formed by all H atoms of the water molecules and the amine groups with all O atoms of the SO_4_^2-^ anion and its symmetric equivalents (Table 1). The complex cations [Mg(H_2_O)_6_]^2+^ and SO_4_^2-^ anions are linked through O—H···O hydrogen bonds into a three-dimensional network, indicating that SO_4_^2-^ anion is a good hydrogen-bonding acceptor. In addition, the amino cations are hydrogen bonded to the SO_4_^2-^ anions through N—H···O hydrogen bonds, which play an important role in stabilizing the crystal structure (Fig. 2).

### Experimental

Commercial 1,4-diazabicyclo[2.2.2]octane (3 mmol), H_2_SO_4_ (3 mmol) and MgSO_4_ (3 mmol) were dissolved in water. The solvent was slowly evaporated in air, affording colorless block-shaped crystals of the title compound suitable for X-ray analysis.

The permittivity measurement shows that there is no phase transition within the temperature range from 100 to 400 K, while the permittivity is 10.2 at 1 MHz at room temperature.

### Refinement

H atoms attached to C and N atoms were positioned geometrically and treated as riding, with C—H = 0.97 and N—H = 0.91 Å and with *U*_iso_(H) = 1.2*U*_eq_(C, N). H atoms of water molecules were located in difference Fourier maps and refined as riding atoms, with O—H = 0.85 Å and *U*_iso_(H) = 1.5*U*_eq_(O). The highest residual electron density was found at 1.18 Å from H1B atom and the deepest hole at 1.42 Å from C2 atom.

### Crystal data

**Table:**

(C_6_H_14_N_2_)[Mg(H_2_O)_6_](SO_4_)_2_	*F*(000) = 928
*M**_r_* = 438.74	*D*_x_ = 1.661 Mg m^−^^3^
Monoclinic, *C*2/*c*	Mo *K*α radiation, λ = 0.71073 Å
Hall symbol: -C 2yc	Cell parameters from 2014 reflections
*a* = 14.968 (3) Å	θ = 3.1–27.5°
*b* = 9.1860 (18) Å	µ = 0.41 mm^−^^1^
*c* = 14.334 (3) Å	*T* = 298 K
β = 117.12 (3)°	Block, colorless
*V* = 1754.2 (8) Å^3^	0.40 × 0.30 × 0.20 mm
*Z* = 4	

### Data collection

**Table:**

Rigaku SCXmini CCD diffractometer	2014 independent reflections
Radiation source: fine-focus sealed tube	1839 reflections with *I* > 2σ(*I*)
graphite	*R*_int_ = 0.022
Detector resolution: 13.6612 pixels mm^-1^	θ_max_ = 27.5°, θ_min_ = 3.1°
ω scans	*h* = −19→19
Absorption correction: multi-scan (*CrystalClear*; Rigaku, 2005)	*k* = −11→11
*T*_min_ = 0.89, *T*_max_ = 0.95	*l* = −18→18
8747 measured reflections	

### Refinement

**Table:**

Refinement on *F*^2^	Secondary atom site location: difference Fourier map
Least-squares matrix: full	Hydrogen site location: inferred from neighbouring sites
*R*[*F*^2^ > 2σ(*F*^2^)] = 0.065	H-atom parameters constrained
*wR*(*F*^2^) = 0.169	*w* = 1/[σ^2^(*F*_o_^2^) + (0.0899*P*)^2^ + 2.4679*P*] where *P* = (*F*_o_^2^ + 2*F*_c_^2^)/3
*S* = 1.26	(Δ/σ)_max_ < 0.001
2014 reflections	Δρ_max_ = 1.30 e Å^−^^3^
116 parameters	Δρ_min_ = −1.08 e Å^−^^3^
0 restraints	Extinction correction: *SHELXTL* (Sheldrick, 2008), Fc^*^=kFc[1+0.001xFc^2^λ^3^/sin(2θ)]^-1/4^
Primary atom site location: structure-invariant direct methods	Extinction coefficient: 0.045 (4)

### Fractional atomic coordinates and isotropic or equivalent isotropic displacement parameters (Å^2^)

**Table:**

	*x*	*y*	*z*	*U*_iso_*/*U*_eq_	
S1	0.61852 (4)	0.24340 (6)	0.11814 (4)	0.0233 (3)	
O1W	0.17086 (15)	0.3991 (2)	0.04379 (17)	0.0371 (5)	
H1WA	0.1091	0.3776	0.0197	0.056*	
H1WB	0.1876	0.4636	0.0913	0.056*	
Mg1	0.2500	0.2500	0.0000	0.0216 (3)	
O2W	0.37984 (14)	0.3255 (2)	0.11889 (15)	0.0352 (5)	
H2WA	0.4372	0.3244	0.1203	0.053*	
H2WB	0.3855	0.3321	0.1805	0.053*	
O1	0.68789 (16)	0.1296 (2)	0.18242 (15)	0.0383 (5)	
O3W	0.23727 (17)	0.1023 (2)	0.10262 (15)	0.0397 (5)	
H3WA	0.2082	0.0203	0.0839	0.059*	
H3WB	0.2606	0.1142	0.1684	0.059*	
O2	0.58702 (19)	0.3346 (3)	0.18142 (17)	0.0490 (7)	
N1	−0.02189 (19)	0.2779 (3)	0.15657 (17)	0.0352 (6)	
H1	−0.0387	0.2783	0.0870	0.042*	
O3	0.52895 (16)	0.1720 (3)	0.03471 (16)	0.0450 (6)	
O4	0.66399 (19)	0.3290 (2)	0.06522 (19)	0.0472 (6)	
C1	0.0764 (3)	0.2074 (5)	0.2138 (3)	0.0538 (9)	
H1B	0.1276	0.2641	0.2066	0.065*	
H1C	0.0748	0.1110	0.1855	0.065*	
C2	−0.1004 (3)	0.1967 (5)	0.1718 (3)	0.0569 (10)	
H2A	−0.1012	0.0955	0.1522	0.068*	
H2B	−0.1660	0.2382	0.1283	0.068*	
C3	−0.0189 (4)	0.4288 (4)	0.1915 (3)	0.0575 (10)	
H3A	−0.0855	0.4713	0.1572	0.069*	
H3B	0.0254	0.4863	0.1734	0.069*	

### Atomic displacement parameters (Å^2^)

**Table:**

	*U*^11^	*U*^22^	*U*^33^	*U*^12^	*U*^13^	*U*^23^
S1	0.0256 (4)	0.0261 (4)	0.0189 (4)	−0.0006 (2)	0.0108 (3)	−0.00084 (19)
O1W	0.0286 (10)	0.0394 (11)	0.0437 (11)	−0.0001 (8)	0.0166 (9)	−0.0157 (9)
Mg1	0.0226 (6)	0.0232 (6)	0.0179 (6)	0.0001 (4)	0.0085 (5)	−0.0008 (4)
O2W	0.0254 (9)	0.0532 (13)	0.0245 (9)	−0.0056 (8)	0.0091 (8)	−0.0064 (8)
O1	0.0425 (11)	0.0411 (11)	0.0247 (9)	0.0132 (9)	0.0095 (8)	0.0013 (8)
O3W	0.0609 (14)	0.0335 (11)	0.0253 (10)	−0.0144 (9)	0.0203 (10)	−0.0001 (8)
O2	0.0632 (15)	0.0555 (15)	0.0317 (11)	0.0207 (12)	0.0247 (11)	−0.0024 (10)
N1	0.0370 (13)	0.0521 (15)	0.0166 (10)	0.0083 (11)	0.0122 (10)	0.0022 (9)
O3	0.0314 (11)	0.0643 (16)	0.0304 (11)	−0.0149 (10)	0.0064 (9)	−0.0022 (10)
O4	0.0637 (15)	0.0410 (13)	0.0503 (13)	−0.0170 (11)	0.0378 (13)	−0.0022 (10)
C1	0.052 (2)	0.073 (2)	0.0464 (19)	0.0296 (18)	0.0312 (17)	0.0097 (18)
C2	0.0463 (19)	0.078 (3)	0.0433 (19)	−0.0272 (19)	0.0174 (15)	−0.0236 (18)
C3	0.080 (3)	0.0389 (18)	0.064 (2)	0.0159 (17)	0.042 (2)	0.0226 (16)

### Geometric parameters (Å, °)

**Table:**

S1—O4	1.458 (2)	O3W—H3WB	0.8502
S1—O2	1.462 (2)	N1—C3	1.467 (5)
S1—O1	1.466 (2)	N1—C1	1.469 (4)
S1—O3	1.482 (2)	N1—C2	1.490 (5)
O1W—Mg1	2.0856 (19)	N1—H1	0.9100
O1W—H1WA	0.8502	C1—C2^ii^	1.513 (5)
O1W—H1WB	0.8500	C1—H1B	0.9700
Mg1—O2W	2.035 (2)	C1—H1C	0.9700
Mg1—O2W^i^	2.035 (2)	C2—C1^ii^	1.513 (5)
Mg1—O3W^i^	2.0728 (19)	C2—H2A	0.9700
Mg1—O3W	2.0728 (19)	C2—H2B	0.9700
Mg1—O1W^i^	2.0856 (19)	C3—C3^ii^	1.506 (8)
O2W—H2WA	0.8499	C3—H3A	0.9700
O2W—H2WB	0.8502	C3—H3B	0.9700
O3W—H3WA	0.8499		
			
O4—S1—O2	111.89 (16)	Mg1—O3W—H3WA	123.8
O4—S1—O1	110.28 (14)	Mg1—O3W—H3WB	125.4
O2—S1—O1	110.80 (12)	H3WA—O3W—H3WB	110.9
O4—S1—O3	106.49 (14)	C3—N1—C1	111.0 (3)
O2—S1—O3	109.00 (14)	C3—N1—C2	109.1 (3)
O1—S1—O3	108.21 (14)	C1—N1—C2	110.7 (3)
Mg1—O1W—H1WA	112.8	C3—N1—H1	108.7
Mg1—O1W—H1WB	134.3	C1—N1—H1	108.7
H1WA—O1W—H1WB	110.8	C2—N1—H1	108.7
O2W—Mg1—O2W^i^	180.00 (17)	N1—C1—C2^ii^	108.5 (3)
O2W—Mg1—O3W^i^	90.55 (9)	N1—C1—H1B	110.0
O2W^i^—Mg1—O3W^i^	89.45 (9)	C2^ii^—C1—H1B	110.0
O2W—Mg1—O3W	89.45 (9)	N1—C1—H1C	110.0
O2W^i^—Mg1—O3W	90.55 (9)	C2^ii^—C1—H1C	110.0
O3W^i^—Mg1—O3W	180.00 (13)	H1B—C1—H1C	108.4
O2W—Mg1—O1W^i^	91.10 (8)	N1—C2—C1^ii^	108.2 (3)
O2W^i^—Mg1—O1W^i^	88.90 (8)	N1—C2—H2A	110.1
O3W^i^—Mg1—O1W^i^	88.14 (9)	C1^ii^—C2—H2A	110.1
O3W—Mg1—O1W^i^	91.86 (9)	N1—C2—H2B	110.1
O2W—Mg1—O1W	88.90 (8)	C1^ii^—C2—H2B	110.1
O2W^i^—Mg1—O1W	91.10 (8)	H2A—C2—H2B	108.4
O3W^i^—Mg1—O1W	91.86 (9)	N1—C3—C3^ii^	108.51 (18)
O3W—Mg1—O1W	88.14 (9)	N1—C3—H3A	110.0
O1W^i^—Mg1—O1W	180.00 (10)	C3^ii^—C3—H3A	110.0
Mg1—O2W—H2WA	125.8	N1—C3—H3B	110.0
Mg1—O2W—H2WB	119.9	C3^ii^—C3—H3B	110.0
H2WA—O2W—H2WB	110.8	H3A—C3—H3B	108.4
			
C3—N1—C1—C2^ii^	64.8 (4)	C1—N1—C2—C1^ii^	65.4 (4)
C2—N1—C1—C2^ii^	−56.5 (4)	C1—N1—C3—C3^ii^	−55.0 (5)
C3—N1—C2—C1^ii^	−57.0 (5)	C2—N1—C3—C3^ii^	67.3 (5)

Symmetry codes: (i) −*x*+1/2, −*y*+1/2, −*z*; (ii) −*x*, *y*, −*z*+1/2.

### Hydrogen-bond geometry (Å, °)

**Table:**

*D*—H···*A*	*D*—H	H···*A*	*D*···*A*	*D*—H···*A*
N1—H1···O3^i^	0.91	1.87	2.734 (3)	157
O1W—H1WA···O3^i^	0.85	1.90	2.751 (3)	179
O1W—H1WB···O1^iii^	0.85	2.01	2.835 (3)	165
O2W—H2WA···O2	0.85	2.00	2.809 (3)	158
O2W—H2WB···O2^iv^	0.85	1.83	2.674 (3)	173
O3W—H3WA···O4^v^	0.85	1.85	2.694 (3)	170
O3W—H3WB···O1^iv^	0.85	1.92	2.769 (3)	177

Symmetry codes: (i) −*x*+1/2, −*y*+1/2, −*z*; (iii) *x*−1/2, *y*+1/2, *z*; (iv) −*x*+1, *y*, −*z*+1/2; (v) *x*−1/2, *y*−1/2, *z*.
